# Physical Exercise Enhances Cognitive Flexibility as Well as Astrocytic and Synaptic Markers in the Medial Prefrontal Cortex

**DOI:** 10.1371/journal.pone.0124859

**Published:** 2015-05-04

**Authors:** Adam T. Brockett, Elizabeth A. LaMarca, Elizabeth Gould

**Affiliations:** Department of Psychology and Princeton Neuroscience Institute, Princeton University, Princeton, NJ, United States of America; Technion - Israel Institute of Technology, ISRAEL

## Abstract

Physical exercise enhances a wide range of cognitive functions in humans. Running-induced cognitive enhancement has also been demonstrated in rodents but with a strong emphasis on tasks that require the hippocampus. Additionally, studies designed to identify mechanisms that underlie cognitive enhancement with physical exercise have focused on running-induced changes in neurons with little attention paid to such changes in astrocytes. To further our understanding of how the brain changes with physical exercise, we investigated whether running alters performance on cognitive tasks that require the prefrontal cortex and whether any such changes are associated with astrocytic, as well as neuronal, plasticity. We found that running enhances performance on cognitive tasks known to rely on the prefrontal cortex. By contrast, we found no such improvement on a cognitive task known to rely on the perirhinal cortex. Moreover, we found that running enhances synaptic, dendritic and astrocytic measures in several brain regions involved in cognition but that changes in the latter measures were more specific to brain regions associated with cognitive improvements. These findings suggest that physical exercise induces widespread plasticity in both neuronal and nonneuronal elements and that both types of changes may be involved in running-induced cognitive enhancement.

## Introduction

Physical exercise is known to enhance cognition in humans across multiple age groups ranging from school age children to the elderly, as well as in healthy individuals and patient populations [1,2]. The types of cognitive tasks known to be improved by physical exercise in humans are varied, including tests of vocabulary, working memory and executive function [3–6]. Consistent with the wide range of improved cognitive function, physical exercise also has widespread effects on human brain structure, increasing volume in many brain regions [1].

Understanding the cellular processes that underlie physical exercise-induced improvements in brain function will help us to understand cognition in general, as well as to identify mechanisms that might be utilized for therapeutic improvement of learning and memory. Toward this end, a considerable effort has been focused at understanding the effects of running on cognitive function in rodent models, although the vast majority of this research has focused solely on the hippocampus [1,2,7]. Studies in both rats and mice have shown that running enhances performance on tasks that require the hippocampus, including context fear conditioning and spatial navigation learning [8–12]. By contrast, relatively few studies in rodents have examined the effects of running on cognitive functions that require brain regions other than the hippocampus.

A large body of evidence suggests that running influences a variety of neuronal measures, including synaptic plasticity, adult neurogenesis and dendritic spine density in the hippocampus [8, 12–15]. By contrast, relatively few studies have considered the possibility that running alters nonneuronal cells in a manner consistent with their involvement in running-induced cognitive enhancement in brain regions important for cognition beyond the hippocampus [16,17]. Astrocytes, the most common nonneuronal cell type in the brain, communicate with both neurons and the brain vasculature [18,19]. Studies indicate that astrocytes play an important role in synaptic plasticity, including long-term potentiation (LTP) [20], suggesting their potential involvement in learning and memory. Indeed, transplantation of human astrocytes into the mouse brain results in improved cognitive performance on a wide range of tasks, potentially through enhancements in synaptic plasticity [21]. Despite these intriguing observations, previous studies have not established whether running-enhanced cognitive improvements are associated with changes in astrocytes. We sought to investigate this possibility as well as to examine whether brain regions that support cognition in addition to the hippocampus were affected by running.

## Methods and Materials

### Ethics Statement

The studies described in this manuscript were approved by the Princeton University IACUC (protocol # 1852, approved June 2014) and conformed to the National Research Council Guide for the Care and Use of Laboratory Animals. Animals were deeply anesthetized with Euthasol and transcardially perfused at the end of these experiments.

### Experimental Animals

Adult male Sprague-Dawley rats were housed in groups of 3 with or without ad lib access to a running wheel for 12 days. This timepoint was selected because it is sufficient to induce dramatic structural alterations in the hippocampus [814]. Running distance was recorded daily from digital counters mounted onto the running wheels (Lafayette Instruments). Rats were housed on a reverse 12 hour light-dark schedule (lights off at 0700), and all behavioral testing occurred between 0900 and 1400. 24 hours after the completion of behavioral testing, rats were deeply anesthetized and then perfused using 4% paraformaldehyde. Fixed brains were then processed for histology and microscopic analysis. Separate cohorts were assessed on either object memory tasks or the attentional set-shifting task. Rats tested on the attentional set-shifting task were food-deprived to 85% body weight over 7 days before the start of testing.

### Object Memory Testing

Runners (n = 18) and sedentary controls (n = 18) were tested on two object memory tasks; object in place, a task known to rely on the medial prefrontal cortex as well as the hippocampus or perirhinal cortex, and novel object preference, a task known to rely on the perirhinal cortex [22]. The sequence of testing was counterbalanced and rats were tested only once within a 24 hour period. Before testing, rats were habituated to an empty testing arena (17 x 17x 17 cm) for 10 minutes/ day for 3 days. On test days, rats were placed in the center of the arena and behavior was scored by an experimenter unaware of the treatment condition. Each task consisted of a 5-minute acclimation period in which animals were exposed to different objects that varied in size (6 x 7 x 10 cm–15 x 17 x 10 cm). During object in place, 4 different objects were placed in each of the 4 corners of the box 3 cm away from the arena walls. For novel object preference, 2 identical objects were placed in 2 corners of the same side of the arena 3 cm away from the walls. For both tasks, following a 5-minute break during which rats were returned to their home cages, rats were again placed in the center of the testing arena for a 3-minute test. During object in place, rats were exposed to the same 4 objects, but the location of 2 of the objects was switched. During novel object preference, animals were exposed to 2 objects, 1 familiar and 1 novel. The sides of the arena in which either the orientation of the objects or the novel object were presented were counterbalanced. The time spent with the novel object or orientation was recorded. A discrimination ratio was calculated as the time spent with the novel object or orientation divided by the total time spent exploring both novel and familiar objects/ orientations during the test phase [22].

### Attentional Set-Shifting Task

A separate cohort of runners (n = 12) and sedentary controls (n = 12) were tested on the attentional set-shifting task, a test of cognitive flexibility [23,24]. Attentional set-shifting consisted of 3 days of testing in which rats learned to discriminate between digging media or the texture covering the digging container to retrieve a food reward (1/4 of a Froot Loop). On testing days, rats were habituated to the testing room for 10 minutes prior to the start of testing. The attentional set-shifting task consists of 5 separate discriminations: simple discrimination (SD), compound discrimination (CD), intradimensional shift (IDS), reversal (REV), and extradimensional shift (EDS). For each phase, rats must reach a criterion of 6 consecutive correct trials of retrieving the food reward before advancing to the next phase. Performance on the reversal is dependent on the orbitofrontal cortex while performance on the extradimensional shift is dependent on the medial prefrontal cortex [23,25].

### Immunolabeling for astrocyte and synaptic markers

Brains for immunohistochemical analysis were postfixed for 48 hours before processing. 40 μm coronal sections (1:12) were cut from half brains into a bath of 0.1 M PBS using a Vibratome. For astrocyte immunolabeling, free-floating sections were rinsed in 0.1 M PBS, pH = 7.5, and incubated with 3% normal donkey serum, and 0.1 M PBS with 0.1% Triton X-100 and either rabbit anti-S100 (1:10,000; Dako) or rabbit anti-aquaporin-4 (1:500; Santa Cruz Biotechnology), and stored at 4°C. S100, an astrocyte specific calcium binding protein, was used to visualize astrocyte cell bodies [26]. Aquaporin-4, a water channel, was used to visualize astrocytic endfeet on blood vessels [27]. After incubation in primary antisera for 24 hours, the sections were rinsed, incubated in donkey anti-rabbit Alexa 488 (1:250; Invitrogen) in the dark for 60 min at room temperature, rinsed and mounted onto Suprafrost Plus slides, and coverslipped using glycerol in TBS (3:1). All sections were counterstained with the DNA dye, Hoechst 33342 (1:100,000; Molecular Probes). To assess the localization of S100 staining in the astrocyte population, additional sections were stained for both S100 as described above and the astrocyte cytoskeletal marker glial fibrillary acidic protein (GFAP) using guinea pig anti-GFAP (1:1000; Advanced Immunochemical). To assess the location of aquaporin-4 staining to that of astrocytic processes and blood vessels, additional sections were incubated in both anti-aquaporin-4 and anti-GFAP or anti-aquaporin-4 and mouse anti-smooth muscle actin (1:50; Santa Cruz Biotechnology). For analysis of synaptic markers, free-floating sections were rinsed in 0.1 M PBS, pH = 7.5, and incubated with 3% normal donkey serum, and 0.1 M PBS with 0.1% Triton X-100 and either mouse anti-postsynaptic density 95 (PSD-95), a postsynaptic marker, (1:200; Millipore) or rabbit anti-synaptophysin, a presynaptic marker (1:500; Santa Cruz Biotechnology), and stored at 4°C. After incubation in primary antisera for 24 hours, sections were rinsed, incubated in donkey anti-mouse Alexa 568 (1:250; Invitrogen) to visualize PSD-95 or incubated in donkey anti-rabbit Alexa 488 (1:250; Invitrogen) to visualize synaptophysin, in the dark for 60 minutes. Sections were mounted and coverslipped as described above.

Slides were coded until completion of the data analysis. Images from the medial prefrontal cortex, orbitofrontal cortex, hippocampus and perirhinal cortex were taken using a Zeiss confocal microscope (LSM 700; lasers: argon 458/488, HeNe 568). Cross-sectional area measurements of 50 S100+ cells per animal per brain region (medial prefrontal cortex, hippocampus, perirhinal cortex, orbitofrontal cortex) were obtained from 20 μm image stacks using ImageJ (NIH). Regions of interest were chosen based on task relevance. Selected cells were located in close proximity to layer 2/3 pyramidal neurons in the medial prefrontal cortex, perirhinal cortex and orbitofrontal cortex. Cells were located in the CA1 region for the hippocampus analysis. Selected cells had to be fully stained and distinct from surrounding labeled cells. Cross-sectional area measurements were taken from z-stacks using ImageJ (NIH) by circling individual cells at their widest point and using the area function. Cross-sectional area covered by aquaporin-4-immunolabeled blood vessels was taken from 1 μm optical sections. All parameters for background subtraction were optimized and held constant throughout the analysis. For PSD-95 and synaptophysin analyses, mean intensity values were collected from 1 μm image stacks and background, determined from adjacent white matter bundles in olfactory bulbs or from the corpus callosum, was subtracted out.

### DiI Impregnation

Brains of runner (n = 9) and sedentary controls (n = 9) were dissected and blocked down the midline. 2–3 lipophilic DiI crystals (Sigma) were implanted into the corpus callosum to backfill layer 2/3 pyramidal neurons in the medial prefrontal cortex [28]. Tissue was incubated at 37°C for 4 weeks. 100 μm coronal sections throughout the medial prefrontal cortex were cut using a Vibratome into a bath of 0.1 M PBS. Sections were mounted with PBS, coverslipped and viewed using a Zeiss confocal microscope (LSM 510; lasers: HeNe 568) with a 40x water objective. All settings (pinhole size, aperture gain, and offset) were initially optimized and held constant throughout the study. For each rat, 5 pyramidal neurons from layer 2/3 in the medial prefrontal cortex were analyzed in 20–50 μm z-stacks by counting spines along secondary and tertiary apical and basal dendrite segments (lengths totaled 50 μm/ dendrite/ neuron). Neurons selected for analysis were fully impregnated, had clearly identifiable secondary and tertiary branches, and were relatively isolated from other neurons. For each brain, 50 dendritic spines were analyzed in detail (25 apical, 25 basal) for length of the spine neck.

### Statistics

Unpaired two tailed Student’s t-tests were performed on each data set (sedentary x runner) except for the attentional set-shifting data, which were analyzed as a mixed factorial ANOVA.

## Results

### Running enhances object memory

**Fig 1:**
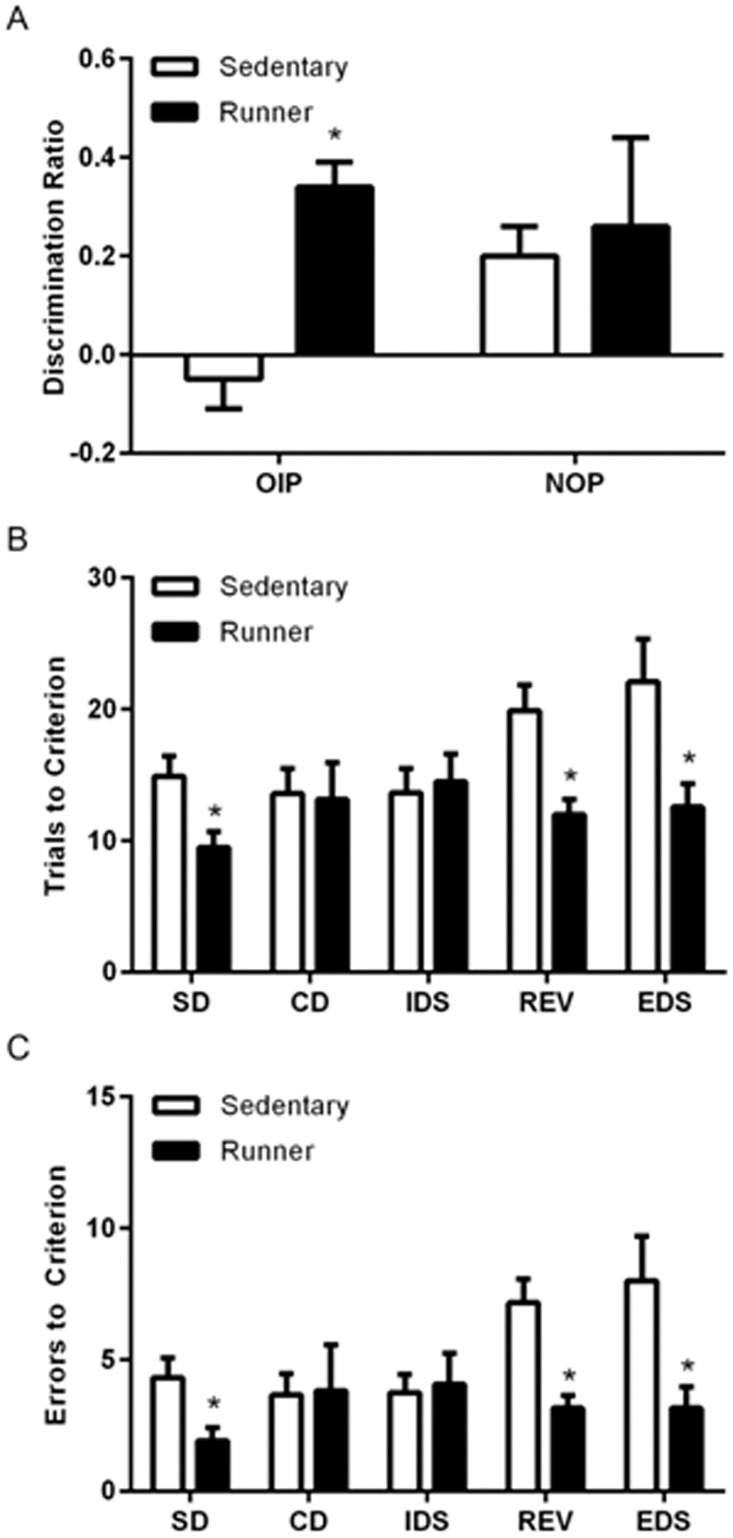
Running enhances cognitive performance on tasks known to require the medial prefrontal cortex and orbitofrontal cortex. *A*, Running enhances performance on the object in place (OIP) task, but not on the novel object preference (NOP) task. *B*, Running results in fewer trials to criterion on the SD, REV and EDS. *C*, Running results in fewer errors on the SD, REV and EDS. Error bars represent SEM. *p<0.05 compared with sedentary rats for A-C. Complex discrimination (CD); intradimensional shift (IDS).

Compared to sedentary controls, runners showed an enhanced discrimination ratio on the object in place task, a medial prefrontal cortex-dependent task (*t*_(30)_ = 5.2, *p* = .001) (Fig 1). It should be noted that sedentary rats did not exhibit the ability to discriminate in the object in place task whereas runners did. The lack of a convincing discrimination ratio among the sedentary rats is likely due to the use of Sprague Dawley rats, a strain that underperforms and shows greater variability on cognitive tasks compared to pigmented strains [29, 30]. Despite the poor performance on the object in place task by sedentary rats, running improved such performance significantly. By contrast, there was no difference in the discrimination ratios of runners and sedentary animals on the novel object preference after 12 days of running (*t*_(27)_ = .839, *p* = .4) (Fig 1).

### Running enhances cognitive flexibility

Runners showed an enhancement on the simple discrimination, reversal, and extradimensional shift of the attentional set-shifting task in both the number of trials to reach criterion (simple discrimination: *t*_(22)_ = -2.77, *p* = .011; reversal: *t*_(22)_ = -3.54, *p* = .002; extradimensional shift: *t*_(22)_ = -2.57, *p* = .017) and the number of errors (simple discrimination: *t*_(22)_ = -2.68, *p* = .014; reversal: *t*_(22)_ = -3.87, *p* = .0008; extradimensional shift: *t*_(22)_ = -2.56, *p* = .018). There was a significant effect of test for both trials to criterion (*F*_(4,116)_ = 2.9, *p* = .03) and errors to criterion (*F*_(4,116)_ = 2.9, *p* = .03) as a well as a significant interaction between condition and test for both trials to criterion (*F*_(4,116)_ = 3.56, *p* = .01) and errors to criterion (*F*_(4,116)_ = 3.825, *p* = .0065) indicating that sedentary rats performed the task as expected, but we are unable to determine whether runners formed an attentional set. These findings may be reflective of runners adopting a different strategy for completing the task in fewer trials and with fewer errors (Fig 1).

### Running enhances astrocyte cell body area

**Fig 2:**
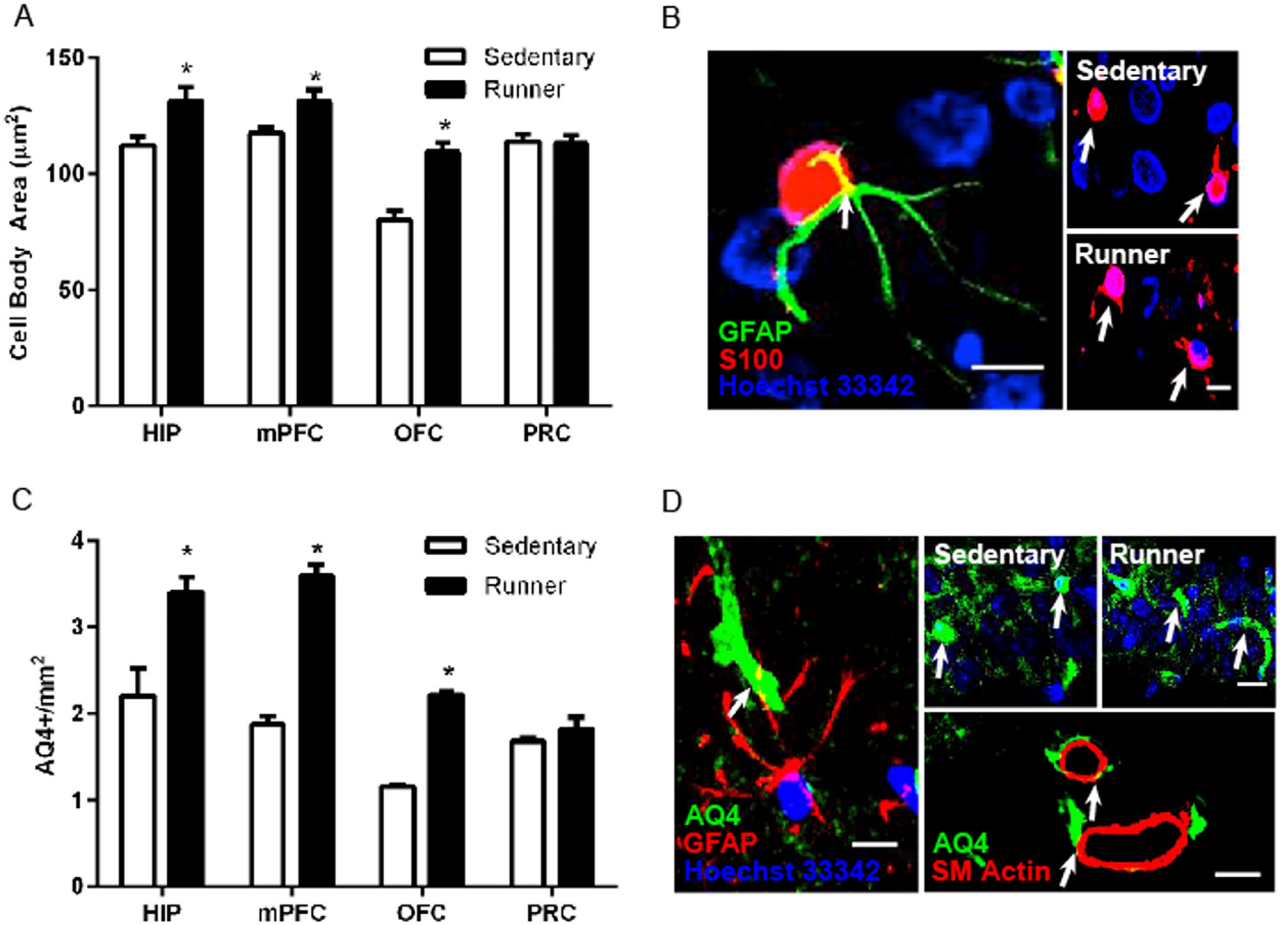
Running alters astrocyte morphology in regions associated with increased cognitive performance. *A*, S100+ astrocyte cell body area is increased in the hippocampus, medial prefrontal cortex, and orbitofrontal cortex. *B*, *Left*: S100+ astrocyte (red) colabeled with GFAP (green). Scale bar = 5 μm. *Right*: Representative images of astrocytes from the medial prefrontal cortex of sedentary and running animals. Scale bar = 10 μm. *C*, Optical density of aquaporin-4, a water channel found in the endfeet of astrocytes, was increased in the hippocampus, medial prefrontal cortex, and orbitofrontal cortex of runners. *D*, *Left*: Aquaporin-4 (green) colabels with GFAP (red). Scale bar = 5 μm. *Right top*: Representative images of aquaporin-4 expression in CA1 of sedentary and running animals. Scale Bar = 20 μm. *Right bottom*: aquaporin-4 labeling (green) is shown in close proximity to smooth muscle actin labeling (red). Scale bar = 20 μm. Error bars represent SEM. *p<0.05 compared with Sedentary for A and C.

Immunolabeling with S100 revealed many cell bodies with proximal processes stained in all brain regions examined. Double labeling of S100 with the astrocytic cytoskeletal marker GFAP revealed co-labeling of many, but not all, S100 cells (Fig 2) with GFAP staining primarily astrocytic processes. We observed an increase in S100-labeled astrocyte cell body area in the hippocampus, medial prefrontal cortex, and orbitofrontal cortex with running compared to sedentary living (hippocampus: *t*_(15)_ = 2.651, *p* = .02, medial prefrontal cortex: *t*_(16)_ = 2.42, *p* = .03, orbitofrontal cortex: *t*_(16)_ = 5.47, *p* = .001). No differences in these measures were observed in the perirhinal cortex between runners and sedentary controls (Fig 2). This pattern of results, with running-induced enhancements in astrocyte markers in hippocampus, medial prefrontal cortex and orbitofrontal cortex, but not perirhinal cortex, is consistent with our behavioral data demonstrating running enhanced performance on cognitive tasks associated with the hippocampus, medial prefrontal cortex and orbitofrontal cortex (object in place, extradimensional shift and reversal phases of the attentional set-shifting task), but not the perirhinal cortex (novel object preference).

### Running enhances expression of the astrocytic water channel marker aquaporin-4

Aquaporin-4 immunolabeling stained blood vessels as well as numerous thin processes in all brain regions examined. Co-labeling with the astrocytic cytoskeletal marker GFAP revealed overlap with aquaporin-4 labeled blood vessels and processes and GFAP-labeled processes (Fig 2). It should be noted that GFAP does not stain astrocytes in their entirety, typically leaving the cell body and distal processes/end feet unstained. Co-labeling with aquaporin-4 and the smooth muscle marker muscle-specific actin revealed close contact between aquaporin-4 labeled astrocytic processes and blood vessels (Fig 2). Optical density measurements of aquaporin-4 staining for the 12 day running experiment revealed a brain region-specific set of changes that was identical to what we observed with S100 cell body area measurements. Compared to sedentary controls, running increased aquaporin-4 optical density in the hippocampus, medial prefrontal cortex, and orbitofrontal cortex, with no differences in the perirhinal cortex (hippocampus: *t*_(16)_ = 3.4, *p* = .004, medial prefrontal cortex: *t*_(16)_ = 10.8, *p* = .0001, orbitofrontal cortex: *t*_(16)_ = 6.57, *p* = .0001) (Fig 2).

### Running enhances dendritic spine density, dendritic spine length and expression of synaptic markers

**Fig 3:**
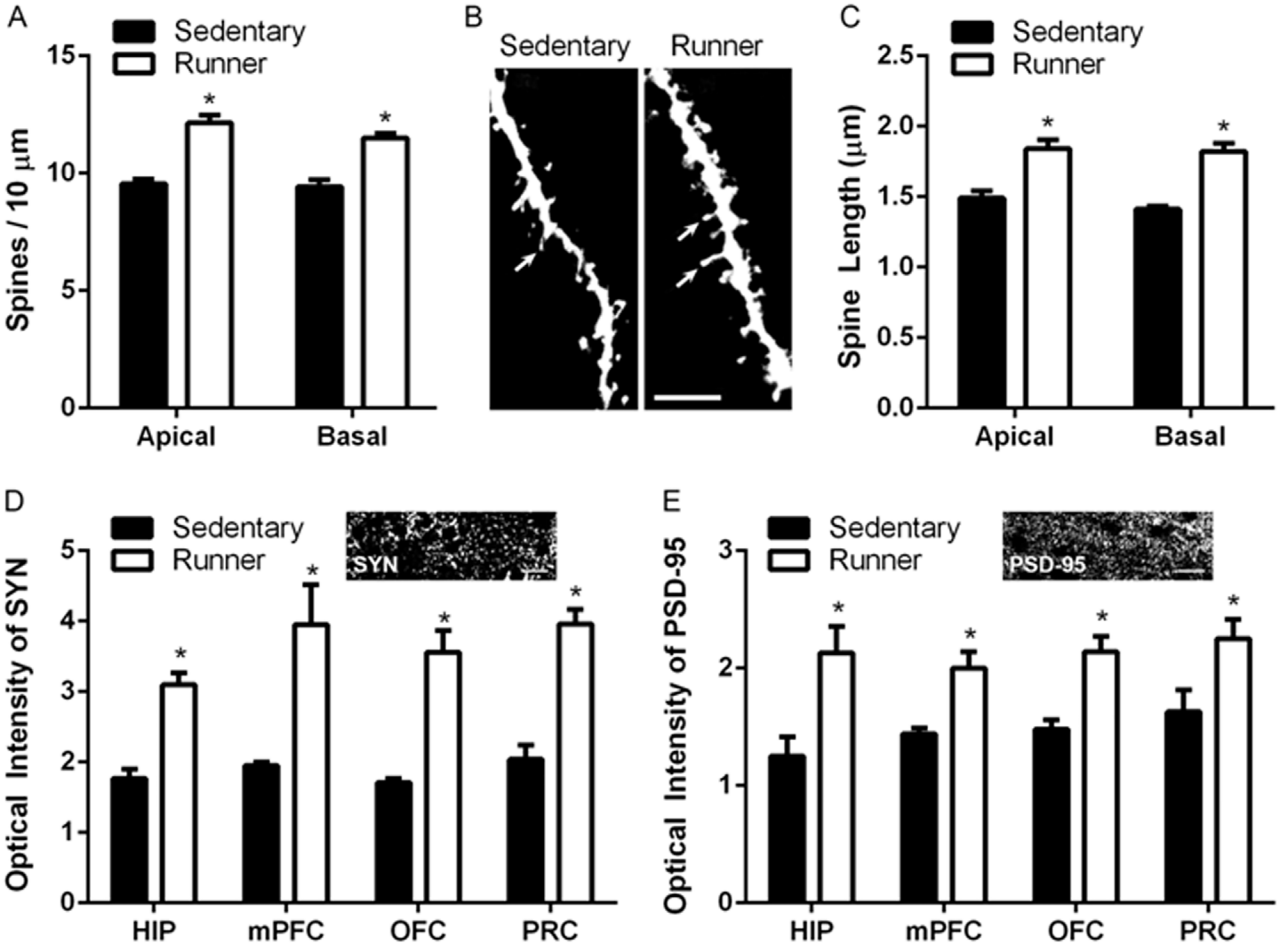
Running increases the number of dendritic spines in medial prefrontal cortex and expression of synaptic markers in several regions supporting cognitive function. *A*, Running increases dendritic spine density on both apical and basal dendrites in the medial prefrontal cortex. *B*, Representative images of DiI labeled layer 2/3 pyramidal neuron apical dendrites in the medial prefrontal cortex and in sedentary and running animals. Scale Bar = 5 μm. *C*, Running increases the average length of spine processes. *D*, Optical intensity analysis of synaptophysin (SYN) reveals increased expression in all regions studied. *Inset*: example of synaptophysin staining in medial prefrontal cortex. *E*, PSD-95 levels are also increased in all regions studied. *Inset*: example of PSD-95 staining in medial prefrontal cortex. Scale Bar = 10 μm. Error bars represent SEM. *p<0.05 compared with sedentary for A, C-E.

Immunolabeling with the presynaptic marker synaptophysin and the postsynaptic marker PSD-95 revealed similar punctate staining in all regions examined (Fig 3). Running resulted in a significant increase in optical intensity for both markers in the hippocampus, medial prefrontal cortex, orbitofrontal cortex and perirhinal cortex compared to sedentary controls (synaptophysin—hippocampus: *t*_(14)_ = 6.27, *p* = .001, medial prefrontal cortex: *t*_(14)_ = 3.5, *p* = .003, orbitofrontal cortex: *t*_(14)_ = 5.8, *p* = .0001, perirhinal cortex: *t*_(16)_ = 6.614, *p* = .001; PSD-95—hippocampus: *t*_(14)_ = 3.13, *p* = .01, medial prefrontal cortex: *t*_(14)_ = 3.5, *p* = .003, orbitofrontal cortex: *t*_(14)_ = 4.33, *p* = .0001, perirhinal cortex: *t*_(16)_ = 2.546, *p* = .02) (Fig 3).

Running increased dendritic spine density on both apical and basal dendrites of layer 2/3 pyramidal neurons (apical: *t*_(13)_ = 6.169, *p* = .0001, basal: t_(13)_ = 6.037, *p* = .0001) in the medial prefrontal cortex (Fig 3). In addition to increasing spine density, the length of individual spines was also significantly increased on both apical and basal trees with running (apical: *t*_(13)_ = 4.162, *p* = .001, basal: t_(13)_ = 6.614, *p* = .00004).

## Discussion

Our results indicate that a moderate duration of running (12 days) enhances performance on cognitive tasks that require the medial prefrontal cortex, such as object in place and the attentional set-shifting task. Similarly, improved performance on a reversal task suggests that orbitofrontal cortex function was augmented as well. When taken together with previous literature showing that performance on cognitive tasks requiring the hippocampus is improved by running [2,7,16,17], our findings suggest that physical exercise has a widespread positive impact on cognition in rodents, as it does in humans [1]. We did not, however, observe an improvement in novel object preference, a task known to require the perirhinal cortex, suggesting that running-enhanced cognitive function may not generalize to all types of learning and memory, at least for the time point examined.

Running is known to increase the density of dendritic spines and numbers of synapses in the hippocampus [13,15]. Our data extend these findings to the medial prefrontal cortex, showing similar dendritic spine and synaptic growth effects after running. We further demonstrate that running enhances the expression of both presynaptic and postsynaptic proteins in the orbitofrontal cortex and perirhinal cortex, in addition to the medial prefrontal cortex and hippocampus, strongly suggesting that this effect does not exhibit a high degree of regional specificity. It seems reasonable to surmise that increased dendritic spine density and increased levels of synaptic proteins in brain regions supporting cognition are likely mediators of running-induced improvements in cognitive function. However, it may be relevant to consider that enhanced expression of both synaptophysin and PSD-95 were observed in the perirhinal cortex and yet, no improvements in function on a task linked to this area, novel object preference, were observed. This suggests that while synaptic enhancements may be involved and in fact, necessary for improved cognition, they may not be sufficient. In this regard, it is important to consider other cellular changes that occur in brain regions linked to running-induced cognitive improvements, such as astrocytic plasticity.

Our findings suggest that astrocyte cell bodies increase in size in response to running and that this effect shows a greater degree of regional specificity than running-induced changes in synaptic proteins, at least for the brain regions we considered in this study. That is, we observed significant increases in S100 labeled cell body area in the medial prefrontal cortex, orbitofrontal cortex and hippocampus, but not in the perirhinal cortex. This regional pattern of results was also observed with running-induced changes in the expression of a marker of astrocytic endfeet in contact with blood vessels, aquaporin-4 (increased expression in medial prefrontal cortex, orbitofrontal cortex and hippocampus, but not in the perirhinal cortex). Taken together with our behavioral results demonstrating improvements on tasks that depend on the medial prefrontal cortex and orbitofrontal cortex (as well as the literature showing improved performance on tasks that depend on the hippocampus) [2,7] and a lack of improvement on a task that depends on the perirhinal cortex, these results raise the possibility that astrocytic changes, in conjunction with synaptic changes, are necessary for the optimization of specific brain regions with physical exercise. It should be emphasized, however, that our results are correlational and do not demonstrate a causal link between astrocytic change and improved cognition.

Astrocytes are a highly heterogeneous and relatively understudied population of cells [19,31]. While it seems that all brain regions in the cortex contain large numbers of astrocytes, regional differences in astrocyte subtypes, function, and even size are thought to exist [19,31]. This makes pinpointing a specific mechanism by which astrocytes may contribute to running-induced increases in various cognitive functions difficult. Astrocytes have been implicated in the regulation of LTP through the release of astrocyte-specific gliotransmitters, such as _D-_serine, as well as in the tight control of glutamate signaling, which is known to modulate neuronal functioning [20,32]. Additionally, astrocytes play a role in the regulation of blood flow and trophic support, both functions that have numerous implications for neuronal functioning and synaptic growth [32, 33]. It is likely that a combination of these mechanisms underlies running-induced changes in cognition, but elucidating their relative roles is limited by currently available technologies [34]. A recent study demonstrated that a reduction in the number of astrocytes alters cognitive performance, but the method used to reduce astrocyte number also adversely affected neurons [35]. Studies designed to address a causal link between astrocytes and cognitive function are needed, but await the development of new approaches or the refinement of existing methods.
